# Medical and Physical Intelligence

**Published:** 1825-10

**Authors:** 


### MEDICAL AND PHYSICAL INTELLIGENCE.

### MORBID ANATOMY.

1. ***Dropsy of the Epiploon, in a Fœtus of eight Months.***—Dr. Ollivier, who relates this case, commencesby remarking that it is now clearly established that the fœtus in utero is subject to nearly the same maladies that are occasionally developed after its birth,—such as phlegmasiæ, &c. and some more particularly connected with the situation of the fœtus in utero. A woman, whose health had been constantly good, was brought to bed, at the end of the eighth month of her pregnancy, of a dead child, but which was well formed. Dr. Ollivier was much struck with the enormous size of the abdomen, the thin parietes of which rendered the fluid which distended it perfectly visible. On opening the abdomen, some spoonsful of a yellow and transparent serum escaped; the peritoneum was colourless, and had no appearance of vessels. The cavity of the abdomen was entirely filled with a transparent tumor formed by the epiploon, the layers of which were separated by a serous, yellow, limpid fluid, contained between them, and in the midst of which flocculi of albumen floated. The surface of the tumor was marked unequally by the vessels which ran in the substance of the epiploon, and which thus formed several irregular, inelastic bridles or bands. The posterior layer was in one spot slightly opaque; the remaining portion possessed its usual transparency. The foramen of Winslow was completely open; and it is presumed that the small quantity of water which escaped from the cavity of the peritoneum had found its way by this aperture. All the viscera of the abdomen were otherwise sound.—(*Archives Generales*, July.)

2. *Encysted Tumors in the Abdomen.*—M. ANDRAL (fils) communicated to the Royal Academy of Medicine the following fact:—A man was admitted into La Charité with a tumor in the abdomen, filling both the hypochondria and the epigastrium. Some months previously he had felt dull pains in the right hypochondrium, and had the jaundice. He died at the end of some time, having suffered by turns the symptoms of phthisis, of enteritis, and peritonitis. On opening the body, tubercles were found in the lungs; a sero-purulent fluid, with false membranes, in the peritoneum; redness of the large intestines; besides the three following diseased appearances:—1. A tumor of the size of a full-grown fœtus; head situated between the right kidney and the concave surface of the liver: it appeared to have originated from the peritoneum; its parietes were fibrous, and it was filled with a purulent fluid, in the midst of which the remains of hydatids were floating. M. Andral believes that this cyst had originally been an hydatid sac, in which hydatids had been successively broken, destroyed, and replaced by pus. The tumor had displaced the liver, which, driven out of the right hypochondrium, filled the epigastric region, and formed a prominence on the left side; besides which, the right lobe of the liver, pressed by the tumor, had suffered a complete atrophy; whilst the left lobe had increased in a very unusual degree. 2. A second cyst, filled equally with broken hydatids, was situated on the course of the biliary vessels, and compressed them. Around and between the broken membranes of the hydatids, there was a quantity of suety matter, similar to that which is sometimes found in ovarian cysts, where it is usually mixed with hair. 3. An old and solid coagulum, which completely obstructed the vena cava inferior, from the origin of the renal vessels to a little above the primitive iliacs. This clot resembled those fibrous layers which fill the sac of aneurisms. The parietes of the vein had suffered a remarkable thickening, as if the result of chronic inflammation: nevertheless, there was not any œdema of the lower extremities.—(*Archives Generales*, August.)

3. *Numerous Calculi in the Bladder.*—M.[n] MURAT recites the case of a man, seventy-five years of age, from whose bladder he removed 678 calculi after death. At the same sitting of the Royal Academy, M. SOUBERBIELLE related several successful operations of lithotomy: one case of the high operation, in a female; another by the lateral operation, the stone weighing five ounces and a half. M. DUBOIS remarks, that the existence of several calculi at the same time lessens very much the benefit of the operations, since it demonstrates a disposition in the kidneys to form them again; and he wishes that the proceedings of M. CIVIALE should be put in practice, for the purpose of breaking down large calculi, even previous to an operation, whether it is to be performed above the pubis or through the perineum. M. RIBES, in confirmation of the first remark of M. Dubois, related the case of a man, who had undergone the operation of lithotomy three times, for the extraction of numerous calculi, and yet at his death, which took place a long time after the last operation, there were three hundred small stones found in the bladder, on opening the body.—(*Revue Medicale*, Juin.)

---

### PATHOLOGY.

4. *Fatal Case of Hemorrhage in the Stomach.*—A patient of mine (says Dr. ELLIOTSON,) last year in St. Thomas's Hospital, whose lungs were somewhat diseased, was sitting up in bed one morning, not particularly ill, when he suddenly vomited some blood, fell back, and died in two or three minutes. The stomach was found distended to the utmost, and, on opening it, an immense coagulum of blood appeared, forming a beautiful mould of the organ. Yet so small was the ruptured vessel, that I could not discover it; nor any more disease of the stomach than a trifling abrasion of the mucous membrane in one spot.—(*Med.-Chirurgical Transactions*, vol. xiii.)

5. *Cause of Barrenness in Prostitutes.*—It is no uncommon thing to see the fimbriated extremity of one or both fallopian tubes adherent to some of the neighbouring parts. In the eighth volume of the Medico-Chirurgical Transactions, p. 505, Mr. LANGSTAFF states that he has noticed this particularly in Cyprians. During the last year two patients who had died in the foul ward of St. Thomas's Hospital, and a notorious prostitute who had died in a clean ward, were opened, and the tubes found adherent; both in two of them, and one in the youngest. We have been accustomed to account for the sterility of prostitutes solely by the supposition of exhausted excitability. It is sufficiently explained by these adhesions, which arise, no doubt, from that state of inflammatory turgescence which occurs to the tubes, in common with all the generative organs, during copulation, and causes them to experience a sort of erection and to embrace the ovaria, being scarcely ever allowed to subside. Not that the occurrence can be universal, because old prostitutes, on marrying after transportation, have occasionally been remarked to breed in New South Wales.—(*Ibid.*)

6. *On the Malignant Pustule of Butchers, &c.*—It is well known that butchers, veterinary surgeons, &c. and all those persons who handle the hides of dead animals, in which the putrefactive process has commenced, are subject to be affected with the malady denominated in this country the *malignant pustule,* unless they take particular precautions to cleanse their hands from the blood, &c. with which they are embued. It appears, from a paper communicated by Dr. BASEDOW, of Merseburg, that this interesting and severe disease has been more than usually common in Germany. Dr. Basedow entitles the disease "die schwarze pocke," the *black pock.*

The first patient was a shepherd, who had lost some sheep by disease, and, on the dissection of one of them, a little serum spurted into his face. The spot thus sprinkled with the fluid of the animal became the primary seat of the disease. He rubbed the part dry with his hand, and thought no more of the circumstance. Six days afterwards he remarked, whilst he was shaving, a little blister upon his cheek. An itching, which was felt in the part, was relieved by dividing this small vesicle. Others appeared on the following day; and the lips of the patient were observed to be considerably thickened. The swelling of these parts soon became very dreadful, and, in three days after the first eruption, there was an extensive gangrene of the cellular membrane. The original point of infection, and around it to the extent of a half-crown, was of a black colour, and the skin perfectly gangrenous. The lips were so considerably thickened, as to project one and three-quarters of an inch from the teeth. The tongue, gums, and throat, were free from swelling or redness. No pain was felt; considerable pressure made upon the swoln parts produced only a dull sensation. Around the part which was gangrenous and tumid, there was considerable redness of the skin, which extended upwards to the forehead, down the neck behind, and, in the anterior part, to the epigastric region. In two days it passed to the scrotum. The fever which usually attends the disease at this period was not present; there was no delirium. The patient was dull, but not deprived of his senses. The return of blood from the head was impeded by the excessive swelling of the neck, which pressed upon the jugular veins and windpipe. Dr. B. prognosticated an attack of apoplexy, unless a considerable diminution of the swelling speedily took place; and the patient died three days after his first visit. It is worthy of remark, that he was throughout perfectly free from any symptoms of gastric derangement.

The second case is that of a Frau Berger, whom Dr. B. found complaining of a bad arm, when he was visiting another patient in the house. Eight days before, a cow belonging to this woman had died. The beast had first lost its appetite, was restless, and sweated copiously. Bleeding was tried, and a few drops of blood during the operation flew upon the woman's arm, and inoculated her with the poison. In two days she felt an unpleasant sense of contraction of the upper arm; and, on the third day, much itching and heat on a small spot. Before any swelling occurred, a small vesicle appeared above the wrist, and others shortly made their appearance near the same part. The lower vesicle only made the rapid progress which characterises the malignant pustule. On the second day after its appearance, it burst; beneath it was a knot of a darkish colour, and surrounded with bluish vesicles. The hand and arm were swelled and inflamed. The axillary glands were affected slightly. Constitutional symptoms followed. The patient ultimately recovered.

It is evident, from this account, that the disease differs but little from that described by ORFILA, under the title of "la pustule maligne." CHAUSSIER and ENNEAUX have also treated of it. The nearer the original point of infection is to the centre of vitality, the greater is the danger. When the face and neck are affected, there is much hazard. The intensity of the morbid poison differs, in common with others,—as, for example, the syphilitic. It is to be lamented that the incipient symptoms of this disease are so slight, as scarcely to attract attention.

According to Dr. B., in the stage of the simple vesicles, caustic washes will be sufficient to destroy the disease; or, perhaps, some irritating dressing may answer the purpose. If the disease has proceeded further, the actual cautery, or the sulphuric or muriatic acids, or the butter of antimony, may be required. A crucial division of the part is recommended at the commencement of the second period. When still further advanced, it will be necessary to cauterise the part previously to the use of the knife, in order to be enabled to divide the parts affected which lie deep. Any means which promote absorption are prejudicial,—as purgatives, bleeding, and emollient applications. Opium will be of service. At the end of the third, and in the fourth period, a different treatment is required. Very free scarifications will be necessary, although we can hope but for little benefit from them if there is very extensive gangrene of the cellular membrane. The external applications should consist of bark, camphor, and spirits. The attendant fever will, of course, demand attention. Stimulating antiseptics, internally given, will prove certainly injurious, before the inflammatory period has run its course.—(GRAFE und WALTHER's *Journal der Chirurgie*, siebenter bund, zweites heft.)

---

### MATERIA MEDICA AND PHARMACY.

7. *On the Ointment of Tartarised Antimony.*—A remarkable effect of the free application of this remedy to the lower extremities, (says Dr. ELLIOTSON,) is the production of pustules upon the genitals. I have ordered this application of it in very many cases of rheumatic pains, and, when Dr. JENNER's work upon its virtues appeared, subjected the legs of a host of patients to its irritation, as in Deacon's case, with the hope of removing the disease of a distant part. In the greater number of examples, the appearance of pustules on the legs, even when the ointment was rubbed entirely below the knees, was accompanied by a crop of pustules chiefly upon the scrotum, but also upon the glans and other parts of the penis, and sometimes in the perinæum, groins, and on the verge of the anus. Similar consequences resulted in females. The friction on the abdomen with the ointment has occasionally had the same effect. I have never noticed the same circumstance when the ointment was rubbed on other parts, unless the patient was careless and dirty, and touched the genitals with it.

It is with the deepest regret that I state my failure with this ointment, employed with the view of curing or lessening the various diseases of distant parts, for which the ever-to-be-revered Jenner hoped that he had found a remedy. But I fully value the ordinary use of the tartarised antimony in the immediate neighbourhood of diseased parts, although convinced that, even in chronic cases, blisters are often of not at all inferior, and often of superior, efficacy.—*(Med.-Chir. Transactions.)*

8. *On the Prussic Acid.*—Dr. ELLIOTSON observes, "I may, perhaps, be permitted here to state, that very extensive experience with prussic acid for four years has fully confirmed all I published respecting it in the work referred to,* and has not furnished me with any additional information, except that it is equally successful against violent and chronic hiccup as against other symptoms of disordered stomach; and that it will in some cases cause ptyalism and irritation of the mouth, as noticed by Dr. MACLEOD."—*(Ibid.)*

---

### SURGERY.

9. *Removal of a great Portion of the Scapula.*—M. JANSON, principal surgeon of the Hôtel Dieu at Lyons, relates the following case:—A female employed in the manufactory of silk, forty-three years of age, began, in 1819, to perceive a tumor on the posterior and middle portion of her shoulder-blade: it was immovable, painful to the touch, and appeared fixed to the bone. In 1824, this tumor had acquired the size of a child's head, was hard, wrinkled, and had occupied all the bone, excepting its inferior border and the supra spinal fossa, and had extended itself into the highest part of the axilla, with an elongated and voluminous pedicle. It was movable in all directions, and carried the arm with it in all the motions given to it. The portion of this swelling which was situated in the axilla, obliged the patient constantly to keep his arm elevated, and at a right angle with the trunk of the body. Violent pains, darting from the shoulder along the arm, were felt in the breast. At length, from want of sleep and indigestion, the patient had become emaciated, and was evidently sinking.

On the 4th of October, M. Janson attempted the removal of the tumor. He included it within two semi-elliptical incisions; dissected the edges of the wound, so as to preserve as much skin as possible, and detached the tumor in every direction; then raising it, and the tumor breaking in the centre throughout its whole thickness, he removed the greatest part; cutting the attachment of the trapezius, of the supra and infra spinatus muscles, he discovered that all that portion of the scapula situated above its spine was in a healthy condition; and, separating by the saw the diseased part of the bone, he thus preserved the articulation of the arm. Finally, laying bare that portion of the tumor situated in the axilla by an oblique incieion from below upwards, he dissected it, and drew it upwards carefully; the cellular tissue which fixed it to the arm gave way, and he succeeded in removing it entirely. All the vessels were then tied; the axilla properly supported by a plug; and the edges of the wound, which was six inches across and nine in length, were brought together with sticking-plaster.

---

* *Numerous Cases illustrative of the Efficacy of the Hydrocyanic or Prussic Acid, in Affections of the Stomach, &c.* 1820.

For a few days the success of the operation was doubtful, from causes affecting the general health; but these were calmed, and the patient quitted the hospital at the end of two months, nearly cured.—On the 15th of March, it was entirely healed, and the motions of the limb were becoming daily more free.—(*Archives Gen.* August.)

10. M. CIVIALE's *Lithontriptor.*—Since the report made by MM. PERCY and CHAUSSIER, in March 1824, relative to the above-named instrument, the author has had under treatment many patients labouring under calculous disorders, which, presenting some remarkable differences, have compelled him to adopt certain modifications in his instrument, and have furnished him with the cases now about to be cited.

The first section contains those cases in which the operation has been rapid and easy: these are six in number. The first is a female, aged seventy-two, worn out with fatigue and pain: the instrument was not introduced with as much facility as was expected; but, when this was done, a stone of about the size of a nut was readily seized; it was very friable; some fragments came away with the urine, others were brought away. Five days afterwards it was decided, after a close examination proved no remains of the stone to exist in the bladder, that recovery was complete. In the second case, two separate introductions of the instrument were necessary. In the first operation, seizing the stone, and bringing away two fragments, was the work of seventeen minutes. The patient was a lieutenant of the navy, and who did not appear to suffer any pain in this first trial. Three days afterwards a second stone was seized and broken, being more friable than the first; and, four days later, the central portion of the first stone was taken out: it was composed of oxalate of lime, covered with uric acid; its diameter was four lines and a half. The third patient required three operations to destroy a calculcus, which the patient had borne for many years. The fourth case required an equal number of operations: there is nothing remarkable in the detail of them; but the family of this patient was remarkable for an hereditary disposition to this disease, both his mother and children having been affected by it. In the fifth case, six operations were requisite, and longer intervals were interposed between them, in consequence of the imprudence of the patient, a captain of chasseurs. Several of the most eminent surgeons of Paris were present at these operations, and bear testimony to the small degree of pain apparently endured by the patient, whose recovery was perfect. The last case, in which the stone was the size of a large nut, required seven introductions of the instrument: there was a slight attack of fever during the treatment of this gentleman, who was of a very irritable constitution.

The second section contains those instances in which the operation has been tedious, and the cure uncertain, without citing the cases, which are long, we shall observe, that many of these patients had been afflicted for several years with large or numerous calculi; that in some there was disease of the bladder or kidneys, with puriform urine; and, in a still smaller number, there was so great a sensibility of the bladder, that the mere passing a catheter was sufficient to bring on alarming symptoms.

M. Civiale, after detailing seven cases of the above description, replies to some objections that have been made to his method, and then draws the following conclusions:—

1st. That his operation is applicable in the majority of cases, where the stone does not exceed one and a half inches in diameter, and where it has not produced too much local or general disease.

2d. That its application is easier, and the cure more certain, in proportion as the disease is more or less recent.

The third reason is only a corollary from the second.

4th. When, from any unforeseen circumstances, lithotomy has not had the desired effect, it does not lessen the chances of the lithoutriptor process.

5th. The introduction of the instrument, and the manœuvres necessary to seize and to break down the calculus, are not attended with much pain, and are not likely to be followed by danger.—(*Revue Medicale*, Juin and Juillet.)

### METEOROLOGICAL JOURNAL,

**By Messrs. William Harris and Co. 50, Holborn, London.**

***From August* 20 *to September* 19, 1825.**

| | MOON. | Rain gauge | THERM. 9 A.M. | THERM. MAX. | THERM. MIN. | BAROM. 9 A.M. | BAROM. 10P.M. | DE LUC'S HYG. 9 A.M. | DE LUC'S HYG. 10P.M. | WIND. 9 A.M. | WIND. 10P.M. | ATMOSPHERIC VARIATIONS. 9 A.M. | ATMOSPHERIC VARIATIONS. 10P.M. | ATMOSPHERIC VARIATIONS. 2 P.M. |
|---|---|---|---|---|---|---|---|---|---|---|---|---|---|---|
| Aug | | | | | | | | | | | | | | |
| 20 | ☽ | | 60 | 72 | 58 | 30.21 | 30.20 | 68 | 75 | NE | NE | Fine | Fine | Fine |
| 21 | | | 66 | 78 | 56 | 30.21 | 30.02 | 78 | 80 | W | W | Cloud. | Sho'ry | Fine |
| 22 | | .10 | 62 | 68 | 60 | 30.18 | 30.10 | 72 | 68 | E | ESE | Cloud. | Fine | Fine |
| 23 | | | 65 | 67 | 53 | 30.08 | 30.00 | 66 | 80 | ESE | SE | Cloud. | Fine | Fine |
| 24 | | | 60 | 72 | 54 | 30.00 | 30.00 | 81 | 79 | ESE | SE | Fine | Fine | Fine |
| 25 | | | 60 | 70 | 54 | 30.00 | 30.02 | 77 | 75 | E | ESE | Fine | Fine | Fine |
| 26 | | | 62 | 70 | 47 | 30.03 | 30.01 | 76 | 74 | ENE | E | Cloud. | Fine | Fine |
| 27 | | | 55 | 60 | 50 | 30.00 | 29.87 | 82 | 94 | ENE | E | Rain | Rain | Cloud. |
| 28 | ○ | .25 | 52 | 63 | 58 | 29.87 | 29.96 | 94 | 79 | NE | E | Rain | Cloud. | Cloud. |
| 29 | | .08 | 62 | 70 | 62 | 29.95 | 29.94 | 86 | 95 | SW | WSW | Rain | Cloud. | Cloud. |
| 30 | | .06 | 66 | 75 | 63 | 29.98 | 29.96 | 92 | 75 | W | SE | Foggy | Fine | Fine |
| 31 | | | 67 | 76 | 59 | 29.99 | 29.96 | 87 | 80 | S | SE | Foggy | Fine | Fine |
| Sep. | | | | | | | | | | | | | | |
| 1 | | | 66 | 72 | 55 | 29.96 | 29.97 | 60 | 70 | SE | NE | Fine | Fine | Fine |
| 2 | | | 68 | 77 | 59 | 30.01 | 30.06 | 68 | 70 | NE | E | Fine | Fine | Fine |
| 3 | | | 62 | 72 | 55 | 30.07 | 29.98 | 74 | 69 | ESE | N | Cloud. | Cloud. | Fine |
| 4 | | | 60 | 72 | 48 | 29.92 | 29.93 | 70 | 67 | N | N | Fine | Fine | Fine |
| 5 | ☾ | | 57 | 62 | 48 | 29.93 | 29.93 | 68 | 70 | N | NNE | Cloud. | Fine | Fine |
| 6 | | | 58 | 65 | 52 | 29.90 | 29.80 | 72 | 68 | NNW | ENE | Cloud. | Fine | Cloud. |
| 7 | | | 56 | 67 | 55 | 29.74 | 29.57 | 75 | 67 | WSW | WSW | Fine | Fine | Fine |
| 8 | | | 60 | 68 | 52 | 29.52 | 29.57 | 75 | 70 | WNW | W | Fine | Fine | Fine |
| 9 | | | 58 | 69 | 57 | 29.62 | 29.62 | 74 | 69 | WNW | WSW | Fine | Fine | Fine |
| 10 | | | 65 | 70 | 59 | 29.57 | 29.40 | 75 | 80 | S | S | Fine | Fine | Rain |
| 11 | | .05 | 65 | 68 | 58 | 29.47 | 29.50 | 75 | 82 | S | SSW | Cloud. | Clou d | Rain |
| 12 | ● | .13 | 63 | 69 | 58 | 29.63 | 29.81 | 78 | 80 | S | SSW | Cloud. | Fine | Fine |
| 13 | | | 65 | 67 | 55 | 29.65 | 29.54 | 86 | 91 | ESE | SSW | Cloud. | Rain | Cloud. |
| 14 | | .16 | 59 | 66 | 59 | 29.41 | 29.32 | 90 | 92 | E | NE | Cloud. | Rain | Cloud. |
| 15 | | .49 | 61 | 66 | 55 | 29.44 | 29.62 | 92 | 86 | N | WNW | Cloud. | Fine | Fine |
| 16 | | | 62 | 69 | 61 | 29.73 | 29.74 | 85 | 87 | WNW | SW | Cloud. | Cloud. | Rain |
| 17 | | 40. | 68 | 72 | 67 | 29.70 | 29.68 | 77 | 79 | SW | WSW | Cloud. | Rain | Cloud. |
| 18 | | | 68 | 70 | 62 | 29.71 | 29.71 | 79 | 80 | SW | SW | Fine | Fine | Cloud. |
| 19 | ☽ | .35 | 67 | 71 | 60 | 29.65 | 29.71 | 80 | 80 | SW | SW | Fine | Fine | Fine |

The quantity of rain fallen in the month of August, was 2 inches and 14.100ths.

